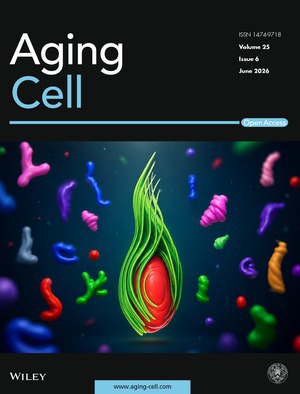# Featured Cover

**DOI:** 10.1111/acel.70585

**Published:** 2026-06-10

**Authors:** Prasanna Katti, Praveena Prasad, Sepiso K. Masenga, Prasanna Venkhatesh, Zer Vue, Andrea G. Marshall, Benjamin Rodriguez, Han Le, Edgar Garza‐Lopez, Alexandria Murphy, Brenita Jenkins, Ashlesha Kadam, Jianqiang Shao, Amber Crabtree, Pamela Martin, Chantell Evans, Mark A. Phillips, David Hubert, Nelson Wandira, Okwute M. Ochayi, Dhanendra Tomar, Clintoria R. Williams, Jennifer Gaddy, Briar Tomeau, LaCara Bell, Taneisha Gillyard, Markis' Hamilton, Vineeta Sharma, Mohd Mabood Khan, Elma Zaganjor, Olujimi A. Ajijola, Estevão Scudese, Tyne W. Miller Fleming, André Kinder, Chandravanu Dash, Anita M. Quintana, Bret C. Mobley, Julia D. Berry, Pooja Jadiya, Dao‐Fu Dai, Annet Kirabo, Oleg Kovtun, Jenny C. Schafer, Sean Schaffer, Renata Oliveira Pereira, Debra D. Murray, Joyonna Gamble‐George, Melanie R. McReynolds, Antentor Hinton

## Abstract

Cover legend: The cover image is based on the article *The MICOS Complex Regulates Mitochondrial Structure and Oxidative Stress During Age‐Dependent Structural Deficits in the Kidney* by Prasanna Katti et al., https://doi.org/10.1111/acel.70534.